# Diversity and abundance of soil macroinvertebrates along a contamination gradient in the Central Urals, Russia

**DOI:** 10.3897/BDJ.10.e76968

**Published:** 2022-02-23

**Authors:** Evgenii Vorobeichik, Alexey Nesterkov, Alexander Ermakov, Maxim Zolotarev, Maxim Grebennikov

**Affiliations:** 1 Institute of plant and animal ecology, UB RAS, Yekaterinburg, Russia Institute of plant and animal ecology, UB RAS Yekaterinburg Russia

**Keywords:** soil macrofauna, earthworms, millipedes, centipedes, spiders, harvestmen, wireworms, ground beetles, rove beetles, molluscs, species diversity, population density, community composition, resistance, forest litter, industrial pollution, heavy metals, copper smelter

## Abstract

**Background:**

Since the late 1980s, long-term monitoring of terrestrial ecosystems in metal-contaminated areas near the Middle Ural Copper Smelter has been carried out in the Central Urals. As a part of these monitoring programmes, the data on species diversity, community composition and abundance of soil macroinvertebrates continue to be gathered.

**New information:**

The dataset (available from the GBIF network at https://www.gbif.org/dataset/61e92984-382b-4158-be6b-e391c7ed5a64) includes a 2004 census for soil macroinvertebrates of spruce-fir forests along a pollution gradient in the Central Urals. The dataset describes soil macrofauna’s abundance (the number of individuals per sample, i.e. the density) and community structure (list of supraspecific taxa, list of species for most abundant taxa and supraspecific taxa or species abundance). Seventeen sampling plots differed in the levels of toxic metal (Cu, Zn, Pb, Cd and Fe) soil contamination from air emissions of the Middle Ural Copper Smelter (heavily polluted, moderately polluted and unpolluted areas). The dataset consists of 340 sampling events (= samples corresponding to upper and lower layers of the 170 soil monoliths) and 64658 rows (2907 and 61751 for non-zero and zero density of taxa, respectively). Arachnida (Araneae and Opiliones), Carabidae (imagoes), Elateridae (larvae), Chilopoda, Diplopoda, Gastropoda, Staphylinidae (imagoes) and Lumbricidae were identified to species level. In contrast, Mermithida, Enchytraeidae, Lepidoptera larvae, Diptera larvae, Hemiptera, Hymenoptera and some other insects were identified to family or order levels. In total, 8430 individuals of soil macroinvertebrates were collected in two soil layers (organic and organic-mineral horizons), including 1046 Arachnida (spiders and harvestmen), 45 Carabidae, 300 Elateridae, 529 Myriapoda, 741 Gastropoda, 437 Staphylinidae, 623 Lumbricidae and 4709 other invertebrates. The presence-absence data on each taxon are provided for each sampling event. An overwhelming majority of such absences can be interpreted as “pseudo-absences” at the scale of sampling plots or study sites. The dataset contains information helpful for long-term ecotoxicological monitoring of forest ecosystems and contributes to studying soil macrofauna diversity in the Urals.

## Introduction

Industrial pollution can drastically affect soil macroinvertebrates (Rusek and Marshall 2000). Soil contamination with toxic metal(loid)s caused by non-ferrous smelters is especially hazardous: some taxa disappear (e.g. earthworms, potworms and molluscs) or decrease in abundance (e.g. centipedes and spiders), leading to the radical transformation of the community structure (Bengtsson and Tranvik 1989, Stepanov et al. 1991, Nekrasova 1993, Nahmani and Lavelle 2002, Gongalsky et al. 2007, Tanasevich et al. 2009, Vorobeichik et al. 2012, Vorobeichik et al. 2019). Such changes drive the slowdown of organic matter decomposition and disintegration of soil aggregates (Korkina and Vorobeichik 2016, Korkina and Vorobeichik 2018), disappearance of some mammals, for example, the common mole (Vorobeichik and Nesterkova 2015, Nesterkova 2019) and the imbalance of mineral nutrients in plants (Sukhareva and Lukina 2014) and birds (Belskii and Grebennikov 2014). Given the considerable role of soil macroinvertebrates in terrestrial ecosystems (Brussaard et al. 2007), they are often used in environmental monitoring and assessment (Cortet et al. 1999, Paoletti et al. 2010).

Areas in the vicinity of point polluters (i.e. sources of atmospheric emissions with an incomparably smaller size than the areas polluted by them) provide a convenient model for analysing the response of terrestrial ecosystems to the toxic load. These areas can be considered the result of a long-term, large-scale natural experiment with ecosystems, which began when a factory was launched. The data obtained in the vicinity of point polluters can be used to reveal the mechanisms of ecosystem resistance and resilience to stress factors (Vorobeichik and Kozlov 2012, Vorobeichik 2022).

We have investigated the response of soil macrofauna communities to industrial pollution in the vicinity of the Middle Urals Copper Smelter. Until recently, this factory was one of Russia's most significant sources of environmental contamination. To date, the study area has been exposed to emissions from the smelter for about 80 years. Toxic metal(loid) concentrations exceed the background levels by several orders of magnitude (Vorobeichik and Kaigorodova 2017, Korkina and Vorobeichik 2018). Amongst other data, information on responses to pollution is available for vegetation (Vorobeichik et al. 2014), soil (Kaigorodova and Vorobeichik 1996, Korkina and Vorobeichik 2016, Korkina and Vorobeichik 2018), soil microflora (Vorobeichik 2007, Mikryukov et al. 2015, Smorkalov and Vorobeichik 2016, Mikryukov and Dulya 2017, Mikryukov et al. 2020), soil microarthropods (Kuznetsova 2009), soil macrofauna (Vorobeichik et al. 2012, Vorobeichik et al. 2019, Vorobeichik and Bergman 2020) and aboveground macro-arthropods (Ermakov 2004, Belskaya and Zinoviev 2007, Belskaya and Vorobeichik 2013, Ermakov 2013, Zolotarev and Nesterkov 2015).

About ten years ago, emissions from the smelter almost ceased, which initiated the natural recovery of adjoining ecosystems. Recent publications were related to the dynamics of metal concentrations in the environment (Vorobeichik and Kaigorodova 2017,Mukhacheva 2017, Belskaya 2018, Nesterkov 2019) and to the natural recovery of humus forms (Korkina and Vorobeichik 2021), vegetation (Vorobeichik et al. 2014, Trubina 2020), leaf-eating insects (Belskaya 2018), birds (Belskii and Lyakhov 2021), epiphytic lichens (Mikhailova 2017, Mikhailova 2020), soil macrofauna (Vorobeichik et al. 2019, Vorobeichik et al. 2020), grass-layer gastropods (Nesterkov and Grebennikov 2020), small mammals (Mukhacheva 2021, Mukhacheva and Sozontov 2021) and the common mole (Vorobeichik and Nesterkova 2015).

Thus, the uniqueness of the study area lies in the ability to investigate in real-time the ecosystem's recovery since there is information about its state before and after reducing emissions in the same sites. Therefore, information on the state of soil macrofauna in 2004 (Vorobeichik et al. 2021) can be taken as a starting point in analysing its recovery. This census is the last before the almost complete cessation of emissions in 2010. A partial analysis of this information has already been presented in a study on species diversity changes along the pollution gradient (Vorobeichik et al. 2012). In addition, an analysis of soil macrofauna recovery at the supraspecific taxa level was carried out (Vorobeichik et al. 2019). Therefore, the advantage of the presented dataset is the ability to implement such an analysis at the species level. In addition, metadata on metal concentrations in forest litter (as an index of toxic load) makes it possible to analyse dose-response dependences and estimate macrofauna resistance thresholds to soil pollution.

We present the sampling-event dataset that introduces the outcomes of a multi-species sampling in the field. Currently, most of the datasets in GBIF are occurrence-based and describe point records of species. In contrast, the contribution of sampling-event datasets remains very low, about 3% of all published datasets (Ivanova and Shashkov 2021, Shashkov et al. 2021). Sampling-event datasets can contain more zeros than non-zeros for the multi-species communities since only a tiny part of the regional species pool may be present in each specific sample. Undoubtedly, the overwhelming majority of such zeros we can qualify as "pseudo-absences" at the scale of sampling plots or, a fortiori, at the scale of study sites.

Nevertheless, such "pseudo-absences" are not needless. These data, given for each species in each sample, provide the most detailed original information about community structure at all investigated spatial scales, from samples to the whole area. Such information is helpful for many research tasks. For example, we can easily estimate the frequency of occurrence at different spatial scales (i.e. within the sampling plot, study site, pollution zone or whole area) for each species or combination of species pooled in ecological groups or supraspecific taxa. Collapsing the data, for example, to the sampling plots scale, will lead to irreversible loss of information, so it is not appropriate. Moreover, if the sampling-event dataset did not contain zeros for "pseudo-absences" of species, we must add them "artificially" for such calculations.

In the context of a pollution gradient study, information about species absence is more crucial than about their presence, assuming that study sites did not differ considerably before a factory operation. The presence-absence data allow the assessment for which species or supraspecific taxa are disappearing with an increase in soil contamination. Considering that most zeros in samples are "pseudo-absences" for taxa, we must apply the taxa absences at least at the scale of sampling plots (after collapsing the data), but not samples for such analysis.

It is important to distinguish the actual disappearance of a species and the declines in a species abundance below the detection limits for the accounting method. Although the interpretation of these two cases is quite different, to differentiate them is a challenging task. Moreover, extraordinary research is needed to detect the pollution-induced elimination of species. For example, we discovered that earthworms and molluscs could inhabit decaying logs in heavily polluted areas near the smelter; however, they were eliminated in topsoils in these sites (Vorobeichik et al. 2020). The presented dataset does not distinguish the species elimination in polluted areas from the declines in their abundance below the detection limits. Nevertheless, the dataset enables us to assess relative changes in species composition and the community structure along a pollution gradient because we used a rigorous sampling design: the number of samples, size of soil monoliths and collecting method were the same in each sampling plot.

## Project description

### Study area description

The study area is situated in the lowest uplands of the Urals (altitudes are 150–400 m a.s.l.) and belongs to the southern taiga subzone. Primary coniferous forests (*Piceaabies*, *Abiessibirica* and *Pinussylvestris*) and secondary deciduous forests (*Betula pendula, Betula pubescens* and *Populustremula*) prevail. Spruce and fir forests with nemoral flora on loam or heavy loam soils dominate on the western slope of the Urals and pine forests on sandy loam or light loam soils prevail on the eastern side. Study sites are located in spruce-fir forests. The ground vegetation layer is dominated by *Oxalisacetosella*, *Aegopodiumpodagraria*, *Gymnocarpiumdryopteris*, *Dryopteriscarthusiana*, *Asarumeuropaeum*, *Maianthemumbifolium*, *Cerastiumpauciflorum* and *Stellariaholostea*. Soil cover is formed mainly by soddy-podzolic soils (Albic Retisols, Stagnic Retisols and Leptic Retisols), burozems (Haplic Cambisols) and grey forest soils (Retic Phaeozems). Zoogenically-active humus form (Dysmull) prevails (Korkina and Vorobeichik 2018, Korkina and Vorobeichik 2021).

The average annual air temperature is +2.0°С; the average annual precipitation is 550 mm; the warmest month is July (+17.7°С) and the coldest month is January (–14.2°С) (mean values for the last 40 years, 1975–2015, according to the data of the nearest meteorological station in Revda). The snowless period is about 215 days (from April to October), the maximum depth of the snow cover being about 40–60 cm.

**Figure 1.**
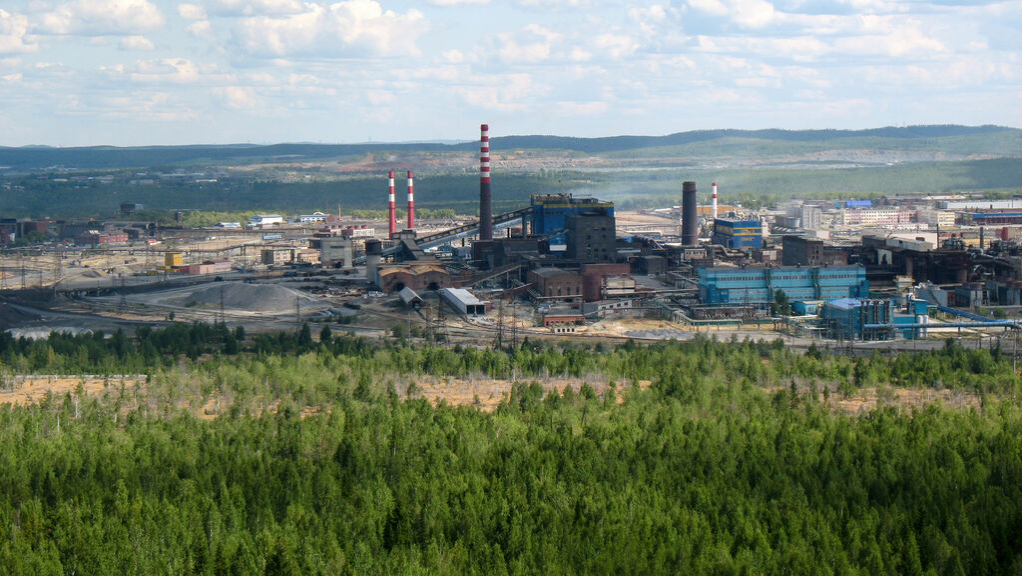
Middle Ural Copper Smelter (MUCS) with adjacent forests heavily impacted by pollution (photo taken in 2012).

The Middle Ural Copper Smelter (MUCS) is located in the suburbs of Revda, 50 km west of Yekaterinburg (Fig. 1). The smelter has been in operation since 1940. The emissions are sulphur dioxide, fluorine, nitrogen oxides and metal(loid)s (Cu, Pb, Zn, Cd, Fe and As). The annual emissions in 1980 reached 225 × 10^3^ t, being reduced to 148 × 10^3^ t in 1990, 63 × 10^3^ t in 2000, 28 × 10^3^ t in 2004 and to only 3–5 × 10^3^ t per year after 2010 (Vorobeichik and Kaigorodova 2017). Current concentrations of heavy metals in the forest litter near the MUCS are very high: Cu, 3500–5500 μg/g; Pb, 2500 μg/g; Cd, 17–20 μg/g; Zn, 600–900 μg/g; i.e. they exceed the background values by factors of 100, 40, 7 and 3, respectively (Vorobeichik and Pishchulin 2016, Korkina and Vorobeichik 2018). Data on pH and metal concentrations in forest litter in the sampling plots are presented later in Table 3.

In the moderately polluted area, emissions have suppressed the tree stand and ground vegetation layer (decreasing species diversity and productivity). Only fragments of the spruce-fir forests have persisted in the heavily polluted area. Near the MUCS, ground-layer vegetation consists of several pollution-tolerant species (*Equisetumsylvaticum*, *Deschampsiacaespitosa*, *Tussilagofarfara*, *Agrostiscapillaris*) and a moss layer has been formed by only one species (*Pohlianutans*). Apart from the metal accumulation and increased acidity, soil transformation manifests itself in the enhancement of the eluvial-gleying process, degradation of soil aggregates, decrease in exchangeable potassium and magnesium, increase in forest litter thickness and shifts from zoogenically-active Mull humus forms to Eumor humus forms without any signs of soil macrofauna activity (Kaigorodova and Vorobeichik 1996, Korkina and Vorobeichik 2016, Vorobeichik and Pishchulin 2016, Vorobeichik and Kaigorodova 2017, Korkina and Vorobeichik 2018, Korkina and Vorobeichik 2021).

## Sampling methods

### Study extent

**Figure 2.**
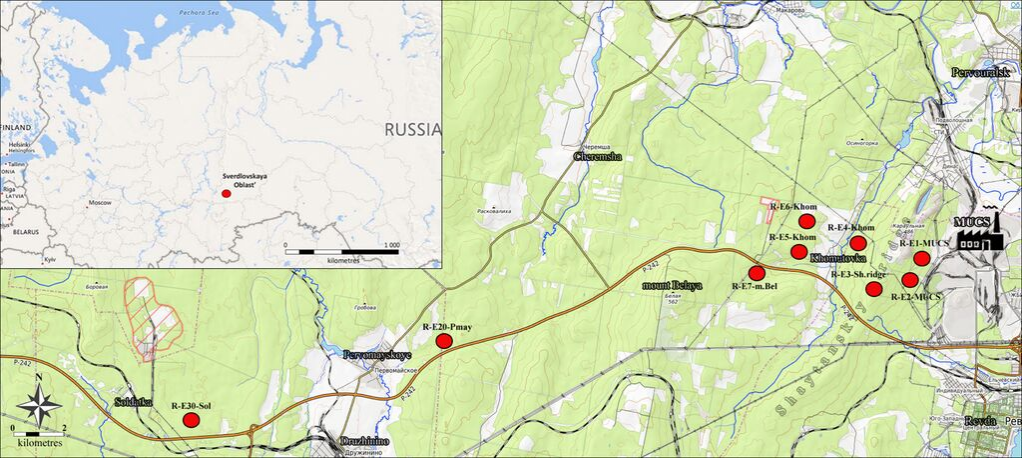
Location of the study sites (= LocationID) in the Central Urals (a scheme based on the data from Open Street Map (OpenStreetMap contributors 2017)).

Study sites (Fig. 2) were located on gentle slopes of ridges in spruce-fir forest. A total of nine study sites (= locationID) were established, corresponding to areas with different pollution levels. The number of sampling plots within each study site ranged from one to three; 20 samples were collected from each sampling plot (Table 1).

The study of soil macrofauna is part of an ongoing long-term monitoring project; the dataset covers the period from 03 July 2004 to 16 August 2004.

### Sampling description

Soil macroinvertebrates were collected in July and August of 2004. Sampling plots 10 × 10 m in size were established in nine study sites (Table 2).

Soil macrofauna was hand-sorted out of soil monoliths 20 × 20 cm in area and 25–30 cm in depth, depending on the presence of macroinvertebrates. The time interval for extracting one soil monolith from the sampling plot was approximately 5 minutes. Ten monoliths were collected from each plot randomly, excluding nearby trunk areas with a radius of 0.5–1 m around large trees (more than 30 cm in diameter) and any visible pedoturbations. During sampling, monoliths were divided into two layers: the O horizon (organic) and A horizon (organic-mineral). Monoliths were placed in plastic bags (separately for the layers), delivered to the laboratory and stored before processing at 12°C for no more than five days (as a rule, 1–2 days). The collected invertebrates were wet-preserved in 70% ethanol; earthworms were carefully washed with water, fixed with 10% formalin and then wet-preserved in 70% ethanol. Ants and relatively large micro-arthropods (springtails, oribatid mites) were not accounted for. A total of 340 samples and 8430 individuals of soil macroinvertebrates were collected.

When preparing the dataset, we assumed that each species recorded in the investigated area could be found in each sample. Based on this assumption, the zero-densities of species in the sample indicated by zero and correspondingly dwc:occurrenceStatus=absent.

To study the metal contents, we collected five pooled samples of forest litter in August 2004 at each sampling plot (85 samples in total, Table 3). Dried samples were ground and sieved (2 mm). The pH was measured potentiometrically (the soil-to-water ratio was 1:25 w/v). We used acid-soluble forms of the potentially toxic metals (Cu, Pb, Cd, Zn and Fe) to approximate its total content and as an index of toxic loads. Metal concentrations were determined by an atomic absorption spectrophotometer AAS 6 Vario (Analytik Jena, Germany) after extraction with 5% nitric acid (HNO_3_) (the soil-to-acid ratio was 1:10 w/v) following USEPA Method 7000B (USEPA 2007).

### Quality control

All soil macrofauna specimens were stored in the Laboratory of Population and Community Ecotoxicology of the Institute of Plant and Animal Ecology, Ural Branch of the Russian Academy of Sciences, Yekaterinburg (IPAE). The specialists of the IPAE performed species identification of most taxa: Maxim P. Zolotarev identified arachnids, millipedes and centipedes; Alexander I. Ermakov identified carabids and elaterids; Maxim E. Grebennikov identified molluscs. Viktor B. Semenov (Institute of Medical Parasitology, Tropical and Vector-borne Diseases named after E.I. Martsinovsky, Moscow) carried out species identification of the staphylinids. Elena V. Golovanova (Laboratory of Invertebrate Systematics and Ecology of Omsk State Pedagogical University) identified earthworms.

## Geographic coverage

### Description

The polygon of study is located in the southern taiga subzone of the Central Urals, 60–70 km westbound from Yekaterinburg. Study sites are placed in spruce-fir forests of non-polluted, moderately polluted and heavily polluted areas in vicinities of the Middle Urals Copper Smelter (MUCS).

### Coordinates

56.785 and 56.905 Latitude; 59.356 and 59.920 Longitude.

## Taxonomic coverage

### Description

General taxonomic coverage is four phyla, seven classes, 16 orders, 39 families, 115 genera and 142 species of soil macroinvertebrates. The species richness of some taxa along a pollution gradient is presented in Table 4.

The community's core in unpolluted and moderately polluted areas is formed by Lumbricidae and Enchytraeidae (30–60% of the total abundance). The earthworm density reached 260 ind./m² (considering cocoons, up to 1000 ind./m²). In total, eight species of earthworms were recorded: two Ural endemics (*Riphaeodrilusdiplotetratheca* (Perel, 1967) and *Pereliatuberosa* (Svetlov, 1924)), an Asian species *Eiseniaatlavinyteae* Perel & Graphodatsky, 1984 and five peregrine species (*Dendrobaenaoctaedra* (Savigny, 1826), *Aporrectodearosea* (Savigny, 1826), *Octolasionlacteum* Orley, 1881, *Bimastosrubidus* (Savigny, 1826) and *Lumbricusrubellus* Hoffmeister, 1843). When approaching the smelter, the abundance of endogeic species sharply decreases (*P.tuberosa* and *A.rosea*), while the epigeic (*D.octaedra*) and epi-endogeic (*R.diplotetratheca*) species are more tolerant to pollution. Earthworm species richness is the same in unpolluted and moderately polluted areas. Earthworms and enchytraeids disappeared in the heavily polluted sites.

Arthropods are represented by arachnids (spiders and harvestmen), myriapods (centipedes and millipedes) and insects. The most diverse and abundant family of arachnids is Linyphiidae (37 species, more than 90% of the total spider population). The dominant spider species are few: *Asthenarguspaganus* (Simon, 1884), *Tapinocybainsecta* (L.Koch, 1869), *Robertuslividus* (Blackwall, 1836) and *Hahniapusilla* C.L.Koch, 1841. No more than a dozen spider species can be classified as subdominants. Harvestmen are scarce (mainly *Nemastomalugubre* (Muller, 1776) and *Oligolophustridens* (Koch, 1836)). In the pollution gradient, the species richness and abundance of arachnids decreases (spiders: from 220 to 30 ind./m², harvestmen: from 12 to 0.5 ind./m²).

The dominant myriapod species are *Lithobiuscurtipes* C.L.Koch, 1847 (Lithobiidae), *Arctogeophilusmacrocephalus* Folkmanova & Dobroruka, 1960 (Geophilidae) and *Polyzoniumgermanicum* Brandt, 1837 (Diplopoda). Myriapod abundance is maximal in the unpolluted sites (up to 220 ind./m² for Chilopoda and 25 ind./m² for Diplopoda) and decreases when approaching the smelter; however, centipedes are common in the heavily polluted sites.

Amongst insects, species identification has been made only for some Coleoptera (Carabidae, Staphylinidae and Elateridae). A total of nine species of ground beetles, 54 species of rove beetles and seven species of click beetles were recorded. The dominant species are few: *Epaphiussecalis* (Paykull, 1790) in Carabidae, *Geostibacircellaris* (Gravenhorst, 1806) and *Mocytafungi* (Gravenhorst, 1806) in Staphylinidae and *Athoussubfuscus* (Muller, 1764) in Elateridae. The abundance and diversity of Carabidae and Staphylinidae decrease when approaching the smelter, while the density of Elateridae did not change.

Eleven species of molluscs were recorded. *Perpolitahammonis* (Strom, 1765) dominates everywhere; subdominant species are *Valloniacostata* (O.F.Muller, 1774), *Discusruderatus* (W.Hartmann, 1821) and *Euconulusfulvus* (O.F.Muller, 1774). Mollusc abundance is maximal in the unpolluted sites (up to 300 ind./m²) and decreases when approaching the smelter. Molluscs disappeared in the heavily polluted areas.

### Taxa included

**Table:** 

Rank	Scientific Name	
phylum	Annelida	
class	Clitellata	
order	Crassiclitellata	
order	Enchytraeida	
phylum	Arthropoda	
class	Arachnida	
order	Araneae	
order	Opiliones	
class	Chilopoda	
order	Geophilomorpha	
order	Lithobiomorpha	
class	Diplopoda	
order	Chordeumatida	
order	Polyzoniida	
class	Insecta	
order	Coleoptera	
order	Diptera	
order	Hemiptera	
order	Hymenoptera	
order	Lepidoptera	
phylum	Mollusca	
class	Gastropoda	
order	Ellobiida	
order	Stylommatophora	
phylum	Nematoda	
class	Enoplea	
order	Mermithida	

## Temporal coverage

**Formation period:** .

### Notes

From 2004-07-03 to 2004-08-16

## Collection data

### Collection name

lepc_soilMacrofauna_2004

### Specimen preservation method

alcohol, formalin

## Usage licence

### Usage licence

Other

### IP rights notes

This work is licensed under a Creative Commons Attribution (CC-BY) 4.0 License.

## Data resources

### Data package title

Diversity and abundance of soil macroinvertebrates along a contamination gradient in the Central Urals, Russia.

### Resource link

https://www.gbif.org/dataset/61e92984-382b-4158-be6b-e391c7ed5a64

### Alternative identifiers

https://ipt.ipae.uran.ru/resource?r=lepc_soilmacrofauna_2004

### Number of data sets

1

### Data set 1.

#### Data set name

Diversity and abundance of soil macroinvertebrates along a contamination gradient in the Central Urals, Russia.

#### Data format

Darwin Core

#### Number of columns

42

#### Download URL

https://ipt.ipae.uran.ru/resource?r=lepc_soilmacrofauna_2004&v=1.4

#### Data format version

1.4

#### Description

The dataset (Vorobeichik et al. 2021) includes a 2004 census for soil macroinvertebrates of spruce-fir forests along a pollution gradient in the Central Urals. The dataset describes soil macrofauna’s abundance (the number of individuals per sample, i.e. the density) and community structure (list of supraspecific taxa, list of species for most abundant taxa and supraspecific taxa or species abundance). Seventeen sampling plots differed in the levels of toxic metal (Cu, Zn, Pb, Cd and Fe) soil contamination from air emissions of the Middle Ural Copper Smelter (heavily polluted, moderately polluted and unpolluted areas). The dataset consists of 340 sampling events (= samples corresponding to upper and lower layers of the 170 soil monoliths) and 64658 rows (2907 and 61751 for non-zero and zero density of taxa, respectively). Arachnida (Araneae and Opiliones), Carabidae (imagoes), Elateridae (larvae), Chilopoda, Diplopoda, Gastropoda, Staphylinidae (imagoes) and Lumbricidae were identified to species level. In contrast, Mermithida, Enchytraeidae, Lepidoptera larvae, Diptera larvae, Hemiptera, Hymenoptera and some other insects were identified to family or order levels. In total, 8430 individuals of soil macroinvertebrates were collected in two soil layers (organic and organic-mineral horizons), including 1046 Arachnida (spiders and harvestmen), 45 Carabidae, 300 Elateridae, 529 Myriapoda, 741 Gastropoda, 437 Staphylinidae, 623 Lumbricidae and 4709 other invertebrates. The presence-absence data on each taxon are provided for each sampling event. An overwhelming majority of such absences can be interpreted as “pseudo-absences” at the scale of sampling plots or study sites. The dataset contains information helpful for long-term ecotoxicological monitoring of forest ecosystems and contributes to studying soil macrofauna diversity in the Urals.

**Data set 1.** 

Column label	Column description
eventID	An identifier for the set of information associated with an Event, constructed from designations of the year, area and habitat of the study, number of the sampling plot, number of the sample and designation of the soil layer. May contain additional information. A variable. Example: "R2004-E1-7-MUCS-61L".
occurrenceID	An identifier for the Occurrence (a row of the "Associated occurrences" data table). Constructed from a combination of dwc:eventID and the number of occurrence within the suggested event. A variable. Example: "R2004-E1-7-MUCS-61L-1".
locationRemarks	Comments or notes about the Location. The investigated areas are subdivided into heavy polluted, moderately polluted and non-polluted; distances from the pollution source (MUCS) are given (in kilometres). A variable. Example: "heavily polluted area | 1 km W from MUCS".
stateProvince	The name of the next smaller administrative region than country (state, province, canton, department, region etc.) in which the Location occurs. A constant "Sverdlovskaya Oblast'".
municipality	The full, unabbreviated name of the next smaller administrative region than county (city, municipality etc.) in which the Location occurs. A variable. Example: "Nizhniye Sergi".
locality	The specific description of the place. Less specific geographic information can be provided in other geographic terms. A variable. Example: "Pervomayskoye".
locationID	An identifier for the set of location information, corresponding to the study sites. A variable. Example: "R-E20-Pmay".
eventDate	The year-month-day of the event. A variable. Example: "2004-07-17".
samplingProtocol	The description of the method or protocol used during an Event. A constant "extraction of soil monoliths followed by hand-sorting in laboratory".
samplingEffort	The amount of effort expended during an Event. A constant "170 soil monoliths in total; 10 monoliths randomly extracted from 10 x 10 m plot on 9 study sites and 17 sampling plots".
sampleSizeValue	A numeric value for a measurement of the size of a sample in a sampling event. A constant "20L x 20W x 25-30D".
sampleSizeUnit	The unit of measurement of the size of a sample in a sampling event. A constant "centimetres".
basisOfRecord	The specific nature of the data record. A constant "PreservedSpecimen".
decimalLatitude	The geographic latitude (in decimal degrees, using the spatial reference system given in geodeticDatum) of the geographic centre of the sampling plot. A variable. Example: "56.7210".
decimalLongitude	The geographic longitude (in decimal degrees, using the spatial reference system given in geodeticDatum) of the geographic centre of the sampling plot. A variable. Example: "59.4280".
coordinateUncertaintyInMetres	The horizontal distance (in metres) from the given decimalLatitude and decimalLongitude describing the smallest circle containing the whole of the Location. A variable. Example: "10".
geodeticDatum	The ellipsoid, geodetic datum or spatial reference system (SRS) upon which the geographic coordinates given in decimalLatitude and decimalLongitude are based. A constant "WGS84".
habitat	A category of the habitat in which the Event occurred. Contains data on the forest stand and soil type of the sampling plots. A variable. Example: "Abieto-picietum nudum on Stagnic Retisol (Cutanic, Toxic)".
lifeStage	The age class or life stage of the invertebrates at the time the Occurrence was recorded. A variable. Examples: "adult", "juvenile", "cocoon" (last one for the earthworms only).
occurrenceRemarks	Comments or notes about the Occurrence. A state of the earthworm cocoons. A variable. Examples: "egg cocoon", "cocoon exuvium".
kingdom	The full scientific name of the kingdom in which the taxon is classified. A constant "Animalia".
phylum	The full scientific name of the phylum or division in which the taxon is classified. A variable. Example: "Annelida".
class	The full scientific name of the class in which the taxon is classified. A variable. Example: "Clitellata".
order	The full scientific name of the order in which the taxon is classified. A variable. Example: "Crassiclitellata".
family	The full scientific name of the family in which the taxon is classified. A variable. Example: "Lumbricidae".
genus	The full scientific name of the genus in which the taxon is classified. A variable. Example: "Dendrobaena".
specificEpithet	The name of the first or species epithet of the scientificName. A variable. Example: "octaedra".
scientificName	The full scientific name, with authorship and date information. A variable. Example: "Dendrobaenaoctaedra (Savigny, 1826)".
scientificNameAuthorship	The authorship information for the scientificName formatted according to the conventions of the applicable nomenclaturalCode. A variable. Example: "(Savigny, 1826)".
taxonRank	The taxonomic rank of the most specific name in the scientificName. A variable. Example: "species".
organismQuantity	A number value for the quantity of organisms.
organismQuantityType	The type of quantification system used for the quantity of organisms. A constant "individuals".
occurrenceStatus	A statement about the presence or absence of a Taxon in the sample. A variable. Examples: "present", "absent". An overwhelming majority of "absences" can be interpreted as "pseudo-absences" at the scale of sampling plots or study sites.
year	The four-digit year in which the Event occurred, according to the Common Era Calendar. A variable. Example: "2004".
month	The ordinal month in which the Event occurred. A variable. Example: "7".
recordedBy	A list (concatenated and separated) of names of people responsible for recording the original Occurrence. A variable. Example: "Maxim E. Grebennikov | Petr G. Pishchulin | Evgenii L. Vorobeichik".
identifiedBy	A list (concatenated and separated) of names of people who assigned the Taxon to the subject. A variable. Example: "Elena V. Golovanova".
country	The name of the country in which the Location occurs. A constant "Russian Federation".
countryCode	The standard code for the country in which the Location occurs. A constant "RU".
ownerInstitutionCode	The name (or acronym) in use by the institution having ownership of the object(s) or information referred to in the record. A constant "Institute of Plant and Animal Ecology (IPAE)".
institutionCode	The name (or acronym) in use by the institution having custody of the object(s) or information referred to in the record. A constant "Institute of Plant and Animal Ecology (IPAE)".
dynamicProperties	A list of additional measurements, facts, characteristics or assertions about the record. The soil layer in which the sample was collected. A variable. Example: "{"soilHorizon":"O"}".

## Figures and Tables

**Table 1.** Total number of sampling plots and samples at the study sites.

Pollution status	Study site	Number of sampling plots	Number of soil monoliths	Number of samples
Heavily polluted area	R-E1-MUCS	1	10	20
	R-E2-MUCS	3	30	60
	R-E3-Sh.ridge	1	10	20
Moderately polluted area	R-E4-Khom	3	30	60
	R-E5-Khom	1	10	20
	R-E6-Khom	1	10	20
	R-E7-m.Bel	2	20	40
Unpolluted area	R-E20-Pmay	2	20	40
	R-E30-Sol	3	30	60

**Table 2.** Characteristics of the sampling plots. Soil description is given according to IUSS Working Group WRB 2015. Soil texture: SL – sandy loam, ML – medium loam, HL – heavy loam, C – clay.

Pollution status	Study site (dwc: locationID)	Sampling plot (Refers to dwc: eventID)	Decimal latitude	Decimal longitude	Soil description	Soil texture of A horizon / lower part of the soil profile	Vegetation
								Unpolluted area	R-E30-Sol	2 (R2004-E30-2…)	56.7996	59.4276	Albic Retisol (Cutanic)	ML / HL	Abietum oxalidosum
3 (R2004-E30-3…)	56.7988	59.4274	Stagnic Retisol (Cutanic)	ML / HL	Abieto-Picietum oxalidosum				
4 (R2004-E30-4…)	56.7985	59.4273	Stagnic Retisol (Cutanic)	ML / HL	Abieto-Picietum oxalidosum				
R-E20-Pmay	15 (R2004-E20-15…)	56.8240	59.5700	Stagnic Retisol (Cutanic)	ML / ML	Picietum oxalidosum		
	17 (R2004-E20-17…)	56.8210	59.5770	Stagnic Retisol (Cutanic)	ML / HL	Picieto-Abietum oxalidosum		
Moderately polluted area	R-E7-m.Bel	1 (R2004-E7-1…)	56.8538	59.7713	Leptic Retisol (Cutanic)	ML / МL	Abietum oxalidosum
		14 (R2004-E7-14…)	56.8528	59.7710	Leptic Retisol (Cutanic)	ML / МL	Abietum oxalidosum
	R-E6-Khom	13 (R2004-E6-13…)	56.8667	59.8000	Stagnic Retisol (Cutanic, Toxic)	ML / МL	Picietum oxalidosum
	R-E5-Khom	6 (R2004-E5-6…)	56.8584	59.7990	Leptic Retisol (Cutanic, Toxic)	SL / ML	Abieto-picietum oxalidosum
	R-E4-Khom	5 (R2004-E4-5…)	56.8518	59.8252	Stagnic Retisol (Cutanic, Toxic)	ML / C	Abieto-picietum oxalidosum
		8 (R2004-E4-8…)	56.8518	59.8293	Stagnic Retisol (Cutanic, Toxic)	ML / C	Picieto-abietum oxalidosum
		9 (R2004-E4-9…)	56.8509	59.8256	Stagnic Retisol (Cutanic, Toxic)	ML / C	Picieto-abietum oxalidosum
Heavily polluted area	R-E3-Sh.ridge	16 (R2004-E3-16…)	56.8444	59.8459	Leptic Retisol (Cutanic, Toxic)	ML / HL	Abietum nudum
	R-E2-MUCS	10 (R2004-E2-10…)	56.8456	59.8637	Stagnic Retisol (Cutanic, Toxic)	ML / C	Picietum nudum
		11 (R2004-E2-11…)	56.8465	59.8629	Stagnic Retisol (Cutanic, Toxic)	ML / C	Picietum nudum
		12 (R2004-E2-12…)	56.8446	59.8648	Stagnic Retisol (Cutanic, Toxic)	ML / C	Abieto-picietum nudum
	R-E1-MUCS	7 (R2004-E1-7…)	56.8462	59.8653	Stagnic Retisol (Cutanic, Toxic)	ML / C	Abieto-picietum nudum

**Table 3.** pH and metal concentrations (mkg/g) in forest litter of the sampling plots. Data are given as mean (standard deviation for n =5).

Pollution status	Study site (dwc: locationID)	Sampling plot (Refers to dwc: eventID)	pH (water)	Cu	Pb	Cd	Zn	Fe
Unpolluted area	R-E30-Sol	2 (R2004-E30-2…)	5.9 (0.2)	43.3 (13.1)	75.4 (18.7)	3.3 (0.5)	309.3 (20.9)	800 (108)
		3 (R2004-E30-3…)	5.3 (0.1)	38.1 (6.7)	71.2 (11.4)	2.6 (0.4)	297.5 (65.8)	1189 (399)
		4 (R2004-E30-4…)	5.5 (0.3)	36.1 (5.5)	82.0 (9.4)	3.1 (0.3)	305.0 (24.7)	979 (227)
	R-E20-Pmay	15 (R2004-E20-15…)	5.6 (0.1)	60.6 (8.8)	99.8 (8.5)	3.6 (0.4)	382.0 (11.9)	1258 (261)
		17 (R2004-E20-17…)	–	56.8 (17.6)	82.0 (9.4)	3.2 (0.4)	210.0 (37.6)	569 (237)
Moderately polluted area	R-E7-m.Bel	1 (R2004-E7-1…)	5.0 (0.2)	647.3 (90.5)	639.5 (63.0)	13.3 (2.0)	747.9 (119.4)	1343 (234)
		14 (R2004-E7-14…)	5.4 (0.4)	454.0 (203.3)	578.0 (153.5)	13.2 (3.0)	818.2 (74.9)	987 (107)
	R-E6-Khom	13 (R2004-E6-13…)	5.1 (0.1)	1523.5 (351.6)	826.9 (153.6)	15.3 (1.2)	846.3 (79.2)	2137 (641)
	R-E5-Khom	6 (R2004-E5-6…)	5.0 (0.1)	1201.2 (321.8)	973.2 (91.4)	19.4 (2.2)	979.1 (165.2)	1918 (762)
	R-E4-Khom	5 (R2004-E4-5…)	4.7 (0.2)	744.9 (205.5)	843.1 (133.0)	8.4 (1.6)	388.0 (74.0)	1259 (441)
		8 (R2004-E4-8…)	5.0 (0.3)	1159.1 (210.2)	1021.6 (196.1)	10.2 (4.2)	510.2 (184.2)	2182 (490)
		9 (R2004-E4-9…)	4.7 (0.1)	1060.9 (179.0)	1052.5 (92.1)	9.2 (1.5)	516.5 (125.1)	2005 (763)
Heavily polluted area	R-E3-Sh.ridge	16 (R2004-E3-16…)	–	2885.9 (821.7)	1175.3 (286.5)	13.2 (3.5)	557.9 (120.6)	3986 (891)
	R-E2-MUCS	10 (R2004-E2-10…)	4.5 (0.1)	2846.3 (509.3)	2057.2 (345.9)	13.9 (5.5)	744.0 (217.0)	8229 (3564)
		11 (R2004-E2-11…)	4.7 (0.1)	2453.0 (366.6)	1907.0 (284.1)	12.4 (3.5)	737.0 (172.4)	6381 (3059)
		12 (R2004-E2-12…)	4.7 (0.1)	2208.2 (520.7)	1567.9 (343.6)	10.2 (4.8)	627.9 (195.4)	6998 (3430)
	R-E1-MUCS	7 (R2004-E1-7…)	4.5 (0.1)	3726.9 (360.7)	1494.2 (242.6)	16.3 (8.0)	693.1 (78.5)	12446 (1190)

**Table 4.** Number of species per sampling plot in the areas differing with soil contamination levels.

Pollution status	Study area	Sampling plot	Taxon								
			Lumbri-cidae	Ara-neae	Opilio-nes	Chilo-poda	Diplo-poda	Cara-bidae	Staphy-linidae	Elate-ridae	Mollusca
Unpolluted area	R-E30-Sol	2	4	6	2	4	1	4	9	1	6
		3	2	7	4	4	1	2	17	1	6
		4	5	8	3	6	2	2	8	4	4
	R-E20-Pmay	15	3	18	0	5	0	2	13	1	3
		17	3	11	1	5	2	0	9	4	2
Moderately polluted area	R-E7-m.Bel	1	4	5	1	3	0	2	7	2	3
		14	4	14	3	3	1	3	14	2	4
	R-E6-Khom	13	2	8	0	3	0	1	8	3	0
	R-E5-Khom	6	2	6	0	3	0	1	9	2	3
	R-E4-Khom	5	1	10	1	1	1	2	7	2	0
		8	1	13	1	4	1	4	3	2	2
		9	2	9	2	3	1	2	9	3	0
Heavily polluted area	R-E3-Sh.ridge	15	0	7	0	2	0	2	8	3	0
	R-E2-MUCS	10	0	4	1	1	0	1	10	5	0
		11	0	2	0	1	0	1	4	2	0
		12	0	1	0	2	0	1	7	4	0
	R-E1-MUCS	7	0	3	0	2	0	1	5	2	0
Unpolluted area total			6	32	5	6	2	6	35	7	8
Moderately polluted area total			6	28	4	4	1	6	30	4	5
Heavily polluted area total			0	9	1	4	0	3	18	6	0
